# Malaria diagnosis and mapping with m-Health and geographic information systems (GIS): evidence from Uganda

**DOI:** 10.1186/s12936-016-1546-5

**Published:** 2016-10-24

**Authors:** Alberto Larocca, Roberto Moro Visconti, Michele Marconi

**Affiliations:** 1 Cosmo Ltd., Accra, Ghana; 2 Università Cattolica del Sacro Cuore, Milan, Italy; 3 Research and Consulting GIS, Natural Resources Management, Marine Ecology, Disaster Risk Reduction, Hue, Vietnam

**Keywords:** Remote diagnosis, Malaria mapping, Mobile phones, Rapid diagnostic tests (RDTs), Process innovation, Healthcare, Information communication technology (ICT), Geospatial health technology, Geographic information systems (GIS)

## Abstract

**Background:**

Rural populations experience several barriers to accessing clinical facilities for malaria diagnosis. Increasing penetration of ICT and mobile-phones and subsequent m-Health applications can contribute overcoming such obstacles.

**Methods:**

GIS is used to evaluate the feasibility of m-Health technologies as part of anti-malaria strategies. This study investigates where in Uganda: (1) malaria affects the largest number of people; (2) the application of m-Health protocol based on the mobile network has the highest potential impact.

**Results:**

About 75% of the population affected by *Plasmodium falciparum* malaria have scarce access to healthcare facilities. The introduction of m-Health technologies should be based on the 2G protocol, as 3G mobile network coverage is still limited. The western border and the central-Southeast are the regions where m-Health could reach the largest percentage of the remote population. Six districts (Arua, Apac, Lira, Kamuli, Iganga, and Mubende) could have the largest benefit because they account for about 28% of the remote population affected by falciparum malaria with access to the 2G mobile network.

**Conclusions:**

The application of m-Health technologies could improve access to medical services for distant populations. Affordable remote malaria diagnosis could help to decongest health facilities, reducing costs and contagion. The combination of m-Health and GIS could provide real-time and geo-localized data transmission, improving anti-malarial strategies in Uganda. Scalability to other countries and diseases looks promising.

## Background

Rapid diagnosis of malaria is difficult, especially in backward rural areas. The gold standard to diagnose malaria is the bright-field microscopy of Giemsa-stained thick and thin blood smears, which allows parasitological confirmation in blood samples and identification of the malaria species. However, light microscopy requires specialized staff and laboratory equipment, not available in forsaken environments. The current alternative to microscopy in remote areas is the rapid diagnostic test (RDT), which detects parasite antigens. RDTs are easier to perform and can be used by operators with less training [1]. However, RDTs have several limitations (such as the impossibility of showing parasite density or detecting persisting antigens once the parasites are cleared) that can lead to misdiagnosis and, therefore, mistreatment [2].

For many reasons rural populations have a preference toward home-based malaria testing [3]. The cost of malaria testing at healthcare facilities is a financial barrier for poor rural households. Travel to the main urban areas where healthcare facilities are mostly located is often difficult and long due to geographical barriers and the lack of good transportation systems. Moreover, shortage of equipment and specialized staff cause congestion at healthcare facilities in the case of disease outbreaks, increasing waiting times for testing and treatment. Many people cannot afford to access healthcare facilities and thus prefer to see if the fever resolves on its own or use RDTs rather than travel to a healthcare spot. Furthermore, the perception of scarce trustworthiness of healthcare facilities equipment and personnel discourage people from accessing appropriate medical structures.

Diffusion of information and communication technologies (ICT) offers unprecedented opportunities to overcome the hindrances to accessing clinical facilities and thus to increase the rate of appropriate treatment of malaria and other infectious diseases. The integration between traditional medical approach and ICT (i.e. e-Health), especially with Smartphone technologies (m-Health), [4] represents a process innovation that could provide cheap, real-time, and geo-referenced data transmission for on-time response to disease outbreaks.

Scientific literature shows the lack of alignment between medical research and ICT developments that can provide cheaply available, but still largely unexploited technological tools. Not much has been done to integrate the several research fields and technologies [5] due also to the inherent interdisciplinary of m-Health [6].

### m-Health technology

Nowadays m-Health technology has gained attention because of the latest developments in medical technology [7]. The application of m-Health on chronic disease outcomes in low- and middle-income countries shows positive impacts [8]. Projects based on m-Health are increasing in Africa and their effectiveness has been proven at several levels, including specialist advice in remote areas [9], drugs supply and stock management, and contribution to national health management [10]. One of the main challenges for m-Health applied to malaria is the reliability of the parasites screening technologies.

Several kinds of m-Health technology and, specifically, of mobile microscopy applications are already described in the scientific literature regarding the detection of soil-transmitted parasite worms [11] or hematologic and infectious diseases [12]. The Cellscope mobile microscope (a mobile phone connected through a frame to a lens) meets the Digital Imaging and Communications in Medicine (DICOM) standards [13]. A reversed-lens mobile phone microscope can generate imaging with little distortion and sufficiently high resolution to be employed for the detection of parasites in blood and stool samples [14]. This solution has excellent potential for further developments especially considering the minimal cost of the apparatus (approximately 30 USD, excluding the mobile phone). Potential application to diagnose malaria was demonstrated through the detection of haemozoin crystals in the blood smear [15]. Therefore, m-Health, and in particular mobile microscopy, can provide a low-cost method of detecting malaria in remote areas, but also could become a precious source of real-time and geo-located data on malaria. Consequently, the integration of m-Health and GIS with existing health systems will boost the chances of identifying the spatial and temporal pattern of malaria and responding accordingly [16]. This will also highlight the environmental [17], climatic [18], and socio-economic [19] risk factors for malaria. As a matter of fact, GIS has become a central component of the vector-borne disease risk-assessment process in public health and epidemiology, and it is recognized as a functional element in the roadmap for malaria elimination [20]. However, the adoption of m-Health technologies in rural areas is subject to the ICT infrastructure bottleneck. In the case of an inadequate ICT backbone, m-Health could widen the existing gap between urban and rural populations.

This paper aims to evaluate theoretically if the ICT infrastructure in a developing setting could be able to support m-Health technology in reaching the portion of the population with limited access to proper health diagnosis. Furthermore, the paper examines the feasibility of m-Health technology for malaria detection in rural settings.

## Methods

This study investigates Ugandan districts where malaria affects the largest number of people and where the application of m-Health technologies, based on the mobile network, has the highest potential impact. The theoretical model to assess the feasibility and the impacts of this technique for malaria detection in developing countries and remote areas requires several types of geographic data. GIS is then used to evaluate the feasibility of m-Health technologies as part of anti-malaria strategies.

The design and the setting of the study are based on:

1. The mobile network coverage to determine in which areas it is possible to use m-Health technologies, as they are based on mobile devices that should be connected to the ICT network to work properly.
2. The combination of geographical models of the human population density with the clinical incidence of malaria, which allows an estimate of the spatial distribution of malaria cases. Overlapping data on mobile network coverage with data on the spatial distribution of malaria cases determines the potential impact of the m-Health technology.
3. An estimation of how many malaria cases covered by the mobile network reside too distant (in terms of travel time) from a healthcare facility. The introduction of m-Health technologies could be initially implemented only in such areas where the benefit is the greatest, thus, where a large number of people affected by malaria do not have immediate access to healthcare facilities.

This model can be applied in every country where data is available and can be extended to other infective diseases such as TB or water-borne parasites. It can be easily implemented using standard GIS software to overlay and analyse different geographical datasets and to produce significant maps and outputs. The present paper focuses on the execution of the feasibility model in Uganda for its characteristics about malaria morbidity and ITC penetration.

### GIS application in Uganda: a case study

According to World Bank statistics [21], Uganda has a population of 37.78 million people (2014), out of which 19.5% are below the poverty line (in 2012, down from 33.8% in 1999). Life expectancy at birth is 59 years. Average monthly temperature (1901–2009) ranges from 23.9 °C in February to 21.6 °C in July, whereas average monthly rainfall peaks in April (149.5 mm) and floors in December (34.4 mm). According to WHO, 48% of the population in 2013 is aged under 15 and only 4% is over 60 [22]. About 85% of the population lives in rural areas.

According to IndexMundi [23], which refers to CIA World Factbook (August 2014), the major infective diseases in Uganda are:

- Food or waterborne diseases: bacterial diarrhoea, hepatitis A and E, and typhoid fever,
- Vector-borne diseases: malaria, dengue fever, and trypanosomiasis-Gambiense (African sleeping sickness),
- Water contact disease: schistosomiasis,
- Animal contact disease: rabies.

However, Malaria is recognized as the leading cause of morbidity in Uganda with 90–95% of the population at risk and it contributes to approximately 13% of the mortality of under five-year-old children [24]. According to the President’s Malaria Initiative (PMI) [25] malaria prevalence in children (up to 59 months) is 42%, but in rural areas in the northern region, this can climb up to 63%. Malaria is highly endemic in most of the country, and Uganda has some of the highest transmission rates in the world. Falciparum is the major source of infection, responsible for 99% of malaria cases. Pyrethroid and carbamate insecticide resistance has been documented in the country. Therefore, malaria places a huge burden on the Ugandan health system accounting for 30–50% of outpatient visits and 15–20% of hospital admissions and 9–14% of patient deaths [25]. The overall malaria-specific mortality is estimated to be between 70,000 and 100,000 child deaths annually in Uganda [26].

The Ugandan national health system is organized at the national level and in district level health centres [25]. At the top of the national health service are the two national referral hospitals, which offer general hospital and comprehensive specialist services, in addition to training and research facilities for medical students. Regional Referral hospitals (14 in total) offer general hospital plus some specialist services. At district level health facilities are organized in general hospitals and four categories of health centres: HC-I, II, III, and IV, which respectively serve at the community, parish, sub-county and county level. Only general hospitals, HC-III and HC-IV have laboratory facilities. HC-II only provide outpatient care and community outreach, while HC-I does not even have physical infrastructure but consist of groups of volunteers (Village Health Teams, VHT) that engage mainly in primary healthcare, prevention campaigns and promoting health services at community level.

In Uganda there are 25.3 million mobile phones, thus a SIM penetration of 64% (2014) and mobile network coverage of 75% of the population and 65% of the land (2013) [27]. Connections, SIM card penetration, and mobile network coverage are growing fast.

To assess m-Health feasibility in Uganda, the authors mapped the distribution of cellular base stations (2G and 3G), the clinical incidence of falciparum, the modelled population density and the travel time to main cities.

The Uganda Communication Commission produced a map of the distribution of 2G and 3G cellular base stations in Uganda [27]. Semi-automatic capture screen software elaborated this map creating a mosaic of images which was assembled and geo-referenced using Esri ArcGIS (version 10.0). The position of both types of the cellular base stations was digitalized. The mobile network coverage was estimated considering that the signal from a base station can cover a radius of approximately 10 km. This assumption was adopted because of the undetermined topographic position and, most important, the undocumented type, brand, and version of the cellular base stations deployed in Uganda. Therefore, using GIS software, a 10 km buffer was mapped around each cellular base station to represent the network coverage.

The modelled clinical incidence of falciparum malaria geo-dataset [28] was multiplied by the population distribution geo-dataset [29] in Uganda using GIS. The output represents the number of persons that are affected by falciparum malaria according to the model per unit of surface.

Two final assumptions are made: first, the equipped healthcare structures are located only in major cities and second, that people would not like to spend more than 1 h to travel to healthcare facilities. The extra time that people spend waiting for testing in the healthcare facility is not considered. Using the global map of accessibility [30] calculations are made about how many patients affected by falciparum malaria live more than 1 h away from a major city and thus are unwilling to travel to the healthcare facilities.

In GIS environment, the zone statistic tool allowed calculating the number of modelled falciparum malaria patients per district, the number of falciparum malaria patients that are within 10 km from the 2G and 3G mobile network, and the number of falciparum malaria patients covered by mobile network residing more than 1 h of travel away from a major city (i.e. healthcare facility equipped with laboratory facilities).

## Results

**Fig. 1:**
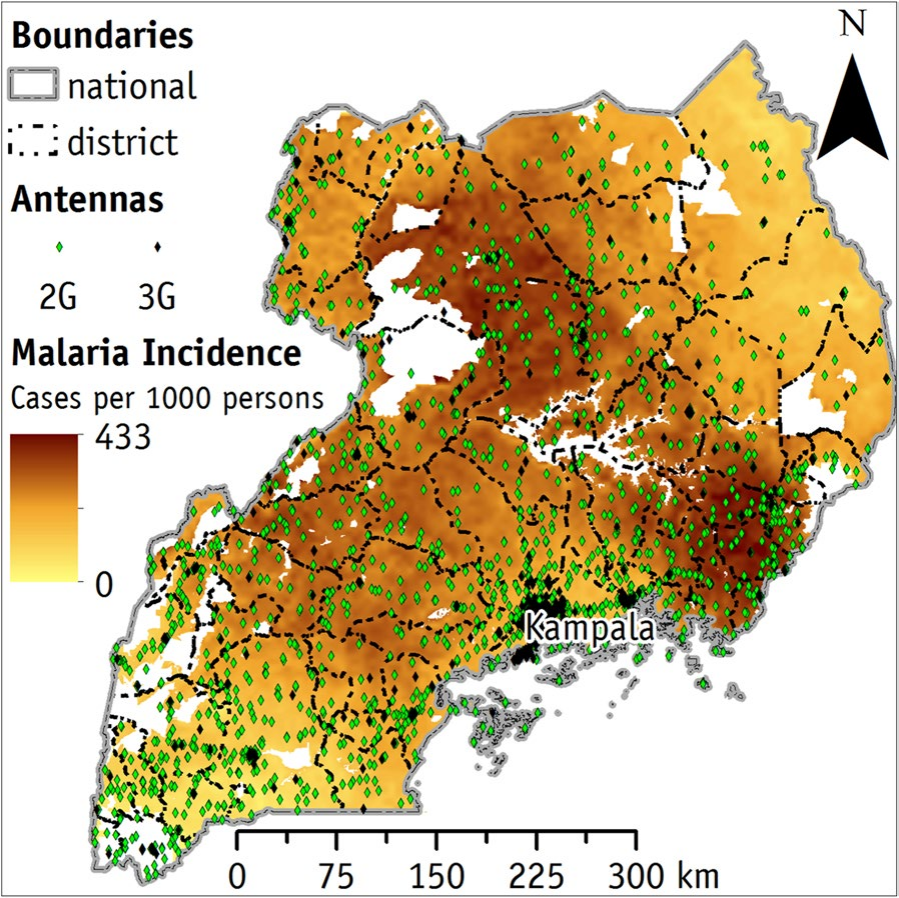
The incidence of falciparum malaria and the distribution of cellular base station in Uganda. Uganda is largely affected by falciparum malaria. Areas with the lowest incidence are the eastern and the south west region. Two-G cellular base station network is diffused in most of the country at the same density, except in the eastern part, where their density is low

Figure 1 shows the distribution of 2G and 3G cellular base stations overlaid with the modelled clinical incidence of falciparum malaria in Uganda. The simple overlay between modelled clinical malaria incidence in Uganda and the cellular base stations shows that the area with a large amount of affected people (in dark brown) are well covered by the 2G cellular base stations (Fig. 1). The eastern region of Uganda has very few cellular base stations, but this area, together with the south-western region, has the lowest malaria incidence values. At a first look, the belt from south–east to north–west (along the Nile River) represents the areas where the highest malaria incidence values are recorded. This belt is well covered by the 2G cellular base stations (green dot).

**Fig. 2:**
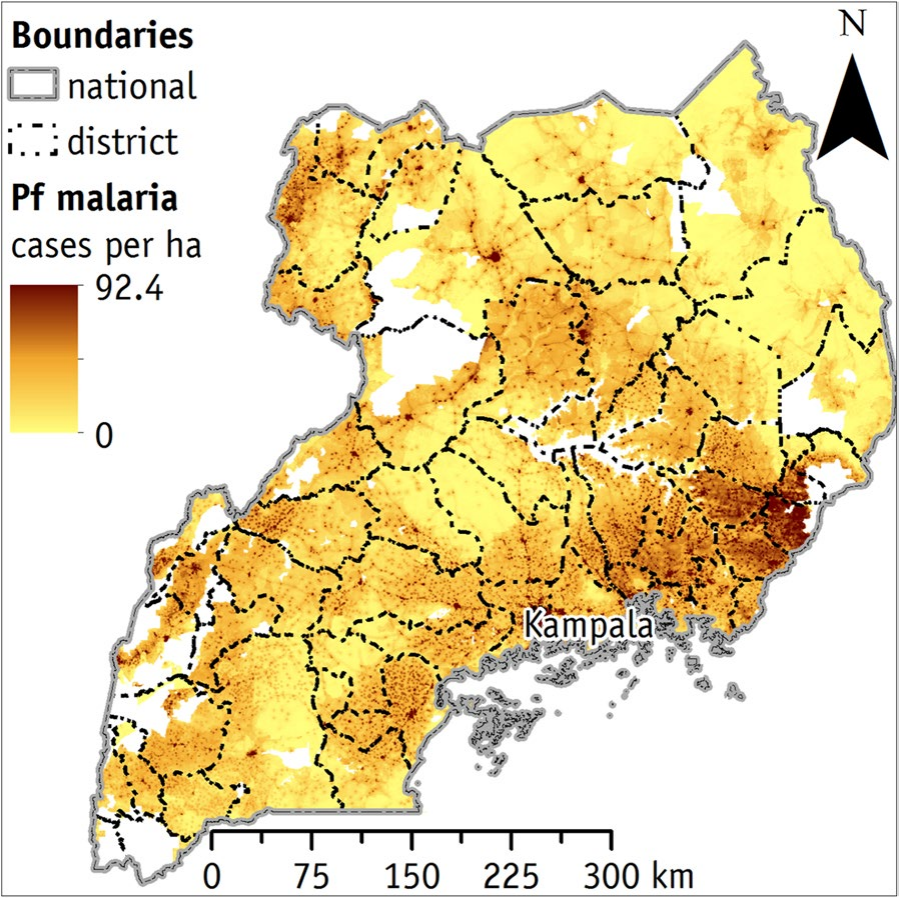
The modelled number of falciparum malaria cases per year and per hectare in Uganda. Integration between the spatial distributions of falciparum malaria incidence (falciparum malaria cases × 1000 people) and of population density model (people × hectare). East region has the lowest number of cases

Figure 2 shows the absolute number of clinical cases of falciparum malaria per year and hectare. The white areas are zones without population, therefore without cases of falciparum malaria. The main falciparum malaria hotspots are the city of Kampala and the south-eastern portion of the country (centred on Mbale district). Secondary hotspots are (1) the Victoria Lake coastal zone, (2) the central part of the country (between Lira, Apac, and Gulu district) and (3) the western border region (from Arua to Kabarole district).

**Fig. 3:**
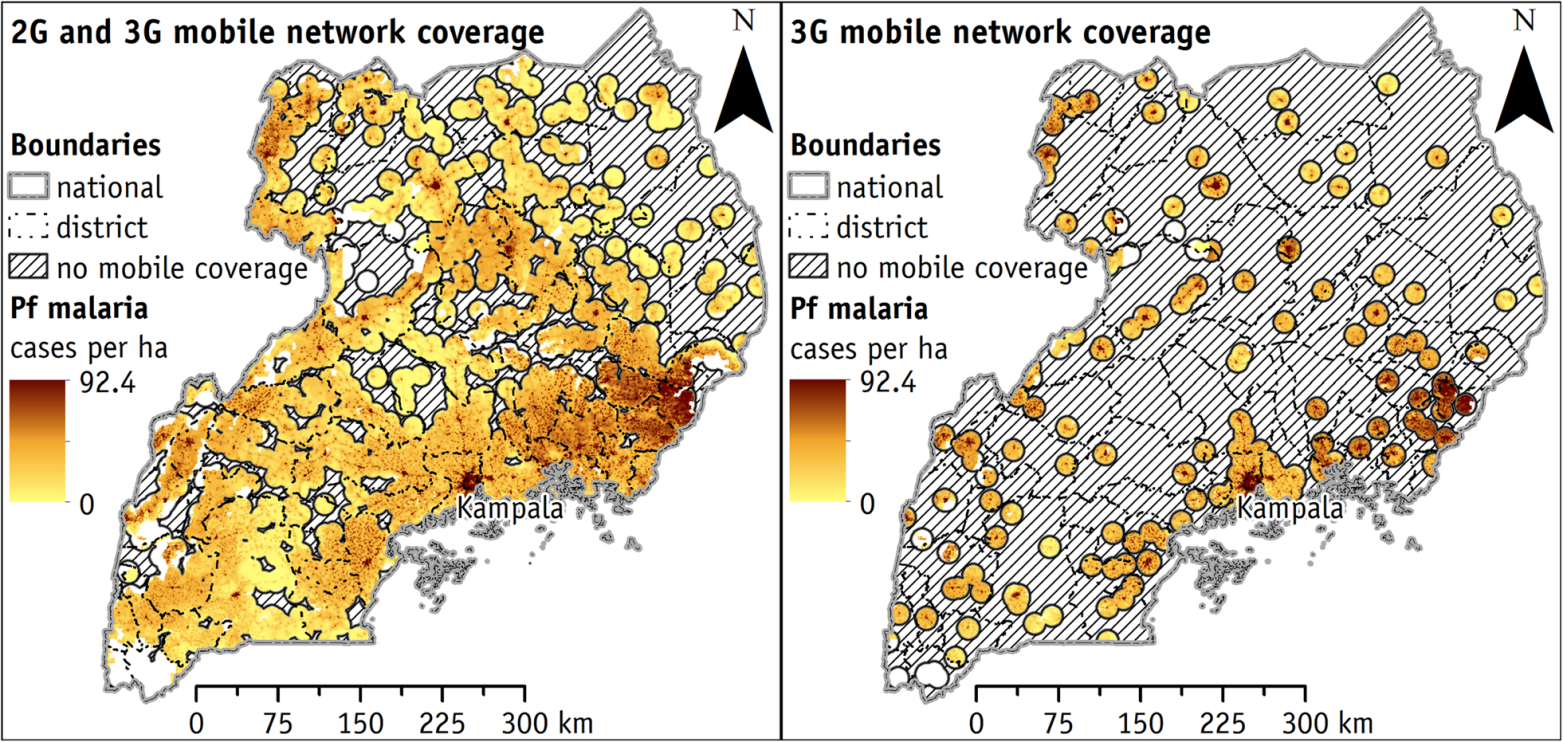
Cases of falciparum malaria covered by mobile network coverage in Uganda. Figure on the *left* represents falciparum malaria cases covered by the 2G cellular base station network. Figure on the *right* shows falciparum malaria cases covered by the 3G cellular base station network. Area without mobile coverage is represented with diagonal hatching

The land surface conjunctly covered by 2G and 3G (Fig. 3) mobile networks corresponds to about 143,000 km^2^. It is about 68% of the surface of Uganda (excluding bodies of water). This percentage is not significantly different from the 65% claimed by the Uganda Communication Commission of land covered by the mobile network in 2013. Therefore this model is the nearest to the mobile network coverage in Uganda and the assumption that each cellular base station can cover an area of 10 km of radius is realistic.

Table 1 summarizes the results of the paper. Specifically, it figures out the potential impact of m-Health adoption on a national scale (first line below table heading) and by district (listed in the first column). In Uganda, there are more than 7.1 million falciparum malaria cases per year (second column) according to the modelled clinical incidence (2015), of which approximately three-quarters (third column) reside more than an hour away from main cities. In the fourth column, the table shows the number of modelled falciparum malaria cases located in areas where there is some mobile network coverage (2G and 3G). They amount to circa 6.6 million cases or 93% (fifth column) of the total number of falciparum malaria cases. Except for a few districts, circled with a red line, the mobile network coverage could reach more than 80% of the falciparum malaria cases. Only three districts have a small area covered by mobile network but they represent only 185,000 cases of falciparum malaria (2% of the total).

**Table 1:** Number of modelled clinical cases of falciparum malaria and cases covered by the mobile network (2G and 3G) by the district

District	Falciparum malaria cases	Falciparum malaria cases 2G and 3G network coverage					Falciparum malaria cases 3G network coverage		
	Total	>1 h	Cases	% Cases	>1 h	% >1 h	Cases	% Cases	>1 h
Uganda	7,111,817	5,248,041	6,620,175	93.1	4,780,161	91.1	3,017,262	42.4	1,719,229
Adjumani	56,340	56,340	42,252	75.0	42,252	75.0	21,624	38.4	21,624
Apac	287,892	275,183	245,277	85.2	233,245	84.8	31,988	11.1	31,843
Arua	254,091	254,091	231,365	91.1	231,365	91.1	125,647	49.4	125,647
Bugiri	114,148	108,314	110,916	97.2	99,089	97.0	35,367	31.0	31,515
Bundibugyo	60,021	51,182	57,696	96.1	49,059	95.9	31,955	53.2	28,379
Bushenyi	142,203	127,916	141,654	99.6	127,382	99.6	73,422	51.6	62,616
Busia	54,652	54,580	54,643	100.0	54,568	100.0	21,754	39.8	21,730
Gulu	192,240	88,992	162,643	84.6	77,286	74.5	91,655	47.7	18,023
Hoima	126,662	126,662	123,371	97.4	123,371	97.4	56,020	44.2	56,020
Iganga	298,180	200,010	293,397	98.4	200,447	97.7	98,568	33.1	49,163
Jinja	91,841	20,358	91,841	100.0	20,358	100.0	77,437	84.3	12,096
Kabale	2649	2649	2647	99.9	2647	99.9	339	12.8	339
Kabarole	101,984	47,464	101,794	99.8	47,281	99.6	63,495	62.3	24,291
Kaberamaido	50,600	34,356	46,006	90.9	30,561	89.0	5198	10.3	3939
Kalangala	8295	7527	7156	86.3	6387	84.8	1584	19.1	1584
Kampala	196,253	0	196,253	100.0	0	0.0	196,253	100.0	0
Kamuli	278,226	235,210	270,785	97.3	227,844	96.9	43,950	15.8	28,516
Kamwenge	81,022	74,970	77,960	96.2	68,907	95.9	26,678	32.9	21,677
Kanungu	26,395	26,395	26,366	99.9	26,366	99.9	12,374	46.9	12,374
Kapchorwa	42,995	36,145	42,243	98.3	35,397	97.9	17,937	41.7	13,456
Kasese	98,293	95,307	97,716	99.4	94,727	99.4	45,447	46.2	44,871
Katakwi	93,144	73,369	73,727	79.2	55,776	76.0	20,417	21.9	10,960
Kayunga	86,261	66,305	79,698	92.4	59,759	90.1	138	0.2	110
Kibale	165,812	165,812	159,074	95.9	159,074	95.9	61,195	36.9	61,195
Kiboga	86,042	86,042	80,506	93.6	80,506	93.6	0	0.0	0
Kisoro	2880	2880	2467	85.7	2467	85.7	561	19.5	561
Kitgum	87,522	87,522	68,621	78.4	68,621	78.4	28,489	32.6	28,489
Kotido	107,843	107,843	57,844	53.6	57,844	53.6	20,081	18.6	20,081
Kumi	163,477	86,452	149,553	91.5	77,849	90.0	86,100	52.7	41,243
Kyenjojo	135,578	117,711	122,328	90.2	99,499	88.8	24,968	18.4	19,687
Lira	249,699	245,238	229,175	91.8	224,723	91.6	76,388	30.6	74,594
Luwero	148,063	102,884	141,360	95.5	96,205	93.5	75,173	50.8	40,975
Masaka	221,199	96,998	219,846	99.4	96,286	99.3	150,699	68.1	55,790
Masindi	174,348	174,348	163,868	94.0	163,868	94.0	104,225	59.8	104,225
Mayuge	66,331	41,338	61,058	92.1	36,035	87.2	8337	12.6	1272
Mbale	312,315	133,592	312,315	100.0	133,503	99.9	229,919	73.6	84,596
Mbarara	188,492	153,222	184,781	98.0	150,986	97.9	59,885	31.8	39,972
Moroto	36,405	36,405	15,808	43.4	15,808	43.4	5712	15.7	5712
Moyo	54,214	54,214	49,703	91.7	49,703	91.7	14,984	27.6	14,984
Mpigi	127,126	97,693	118,941	93.6	89,674	91.8	50,616	39.8	31,608
Mubende	242,633	230,370	231,000	95.2	218,753	95.0	56,582	23.3	52,350
Mukono	174,959	79,240	167,137	95.5	68,708	90.5	112,335	64.2	31,737
Nakapiripirit	40,738	40,738	24,746	60.7	24,746	60.7	8077	19.8	8077
Nakasongola	46,156	46,156	40,230	87.2	40,230	87.2	10,098	21.9	10,098
Nebbi	139,188	139,188	133,317	95.8	133,317	95.8	46,020	33.1	46,020
Ntungamo	46,067	44,328	44,937	97.5	43,194	97.4	11,862	25.7	10,513
Pader	108,060	106,295	79,466	73.5	75,982	73.4	23,619	21.9	23,619
Pallisa	262,041	145,323	251,115	95.8	135,556	93.3	105,524	40.3	57,139
Rakai	104,828	88,756	98,943	94.4	83,272	93.8	47,494	45.3	33,317
Rukungiri	40,408	40,408	39,908	98.8	39,908	98.8	19,343	47.9	19,343
Sembabule	57,389	45,351	53,212	92.7	41,438	91.4	21,028	36.6	15,375
Sironko	98,985	71,724	96,646	97.6	68,413	96.8	10,987	11.1	5612
Soroti	139,009	54,074	124,553	89.6	44,154	85.4	52,332	37.6	10,229
Tororo	272,450	153,936	266,788	97.9	150,779	96.7	186,946	68.6	92,483
Wakiso	185,698	27,160	185,698	100.0	27,160	100.0	166,175	89.5	15,301
Yumbe	81,479	81,479	67,822	83.2	67,822	83.2	42,260	51.9	42,260

**Fig. 4:**
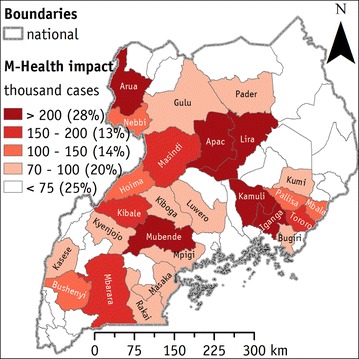
Percentage of m-Health potential cases, i.e. falciparum malaria among people living in remote areas covered by some mobile network. Districts where the implementation of m-Health (i.e. Cellscope mobile) may reach the largest impacts (thousands of potential beneficiaries). Percentages in *brackets* indicate the portions of the potential beneficiaries cumulated in each class

In Uganda 4.78 (sixth column) out of 5.25 (third column) million people with falciparum malaria who live in remote areas (1 h away from a major city) have the availability of some mobile network coverage (2G and 3G). This corresponds to about 91% of the remote population (seventh column) at a national scale. Specifically, at the district level, only six districts, namely Kotido, Moroto, Nakapiripirit Adjumani, Gulu, and Pader, have 75% or less of the falciparum malaria affected population covered by the mobile network. In all remaining districts, the 2G and 3G mobile networks cover the vast majority of the modelled falciparum malaria cases even among remote populations. Figure 4 suggests the districts in which the implementation of m-Health technologies could have the greatest effect to supply malaria diagnosis to remote populations. The mapped indicator quantifies the number of cases (i.e. people infected by falciparum malaria, living in remote areas and covered by some mobile network) that could take potential benefit from the introduction of m-Health technologies.

Six districts (Arua, Apac, Lira, Kamuli, Iganga, and Mubende) have more than 200,000 cases each one that could be approached using m-Health technologies. They represent about 28% of the total m-Health potential cases in Uganda. An additional 13% of m-Health potential cases is localized in four districts (Masindi, Kibale, Tororo, and Mbarara) where there are between 150,000 and 200,000 of m-Health potential cases per district. Further, between 14 and 20% of m-Health potential cases are in other five to eleven districts. Lastly, 30 districts, mapped in white, have less than 75,000 of m-Health potential cases per district, and they amount to 25% of the total m-Health potential cases in Uganda.

The 3G mobile network coverage currently available in Uganda is limited. About three million (eighth column) falciparum malaria cases are in the areas covered by the 3G network, which is 42% (ninth column) of all modelled falciparum malaria cases. Under present conditions, few districts (marked in red in the ninth column: namely Jinja, Kampala, Mbale, and Wakiso) have the vast majority of modelled falciparum malaria cases covered by the 3G mobile network. The 3G network is still in its launch phase and can support m-Health technology only in few Ugandan districts. The last (tenth) column reports the number of falciparum malaria cases among the remote population with available 3G mobile network coverage.

## Discussion

This paper highlights that ICT infrastructures and technologies have the potential to aid public health managers, in particular becoming a pivotal tool for the anti-malarial strategies in a developing country. Uganda was selected as a case study to demonstrate the feasibility of the application of ICT to malaria control and prevention. Specifically, m-Health technologies, based on 2G mobile network, are currently applicable almost everywhere in Uganda. Unfortunately, the current 3G network is not sufficient to support a mass application of m-Health technologies.

In remote areas where only the 2G mobile network is available, however, mobile microscopy could be conveniently used in offline mode if the Smartphone is equipped with automated cell-count software, which would enable field operators to diagnose malaria without a remote consultation through m-Health. In this case, the diagnosis would be instantly made on site at the remote point-of-care while the patient’s record would be transmitted to a larger health centre when faster (3G) connectivity is restored.

### From traditional to the integrated m-Health/GIS malaria healthcare system

**Fig. 5:**
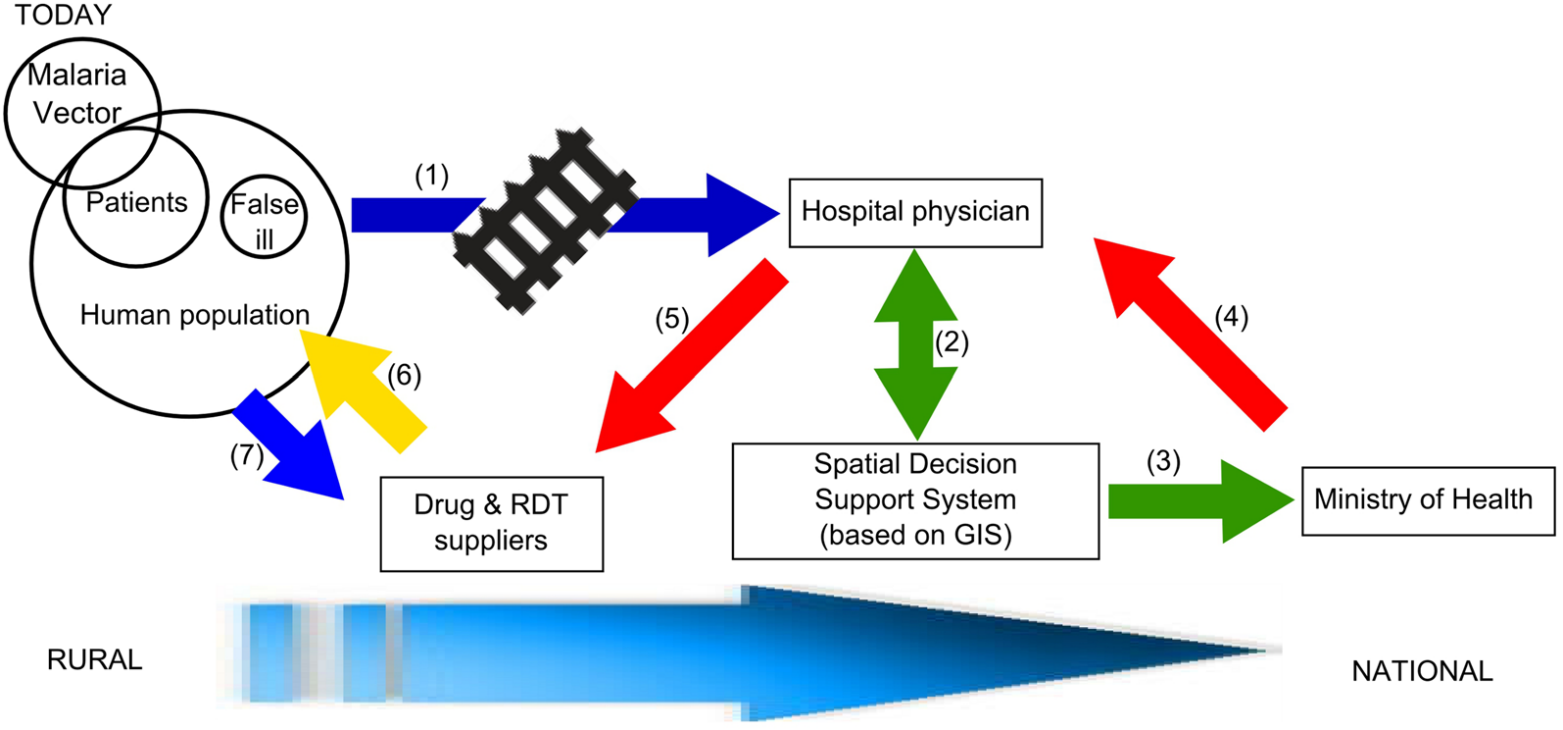
Current malaria management process. Actual malaria management system in Uganda. *Blue arrows* represent physical movement, *red arrows* decision fluxes, *green arrows* information and data flow, and *yellow arrow* drugs supply. Each step (numbers in *brackets*) is described in the text

The implementation of m-Health will drastically transform the public health management system. Figure 5 represents a traditional malaria management system (blue arrows represent physical movement, red arrows decision fluxes, green arrows information and data flow, and yellow arrows drugs supply).

Patients with malaria symptoms reach a healthcare facility where trained medical staff can diagnose malaria (step 1 in Fig. 5). However, there are some barriers to adequate diagnosis and treatment: (a) remoteness of villages and lack of reliable transportation infrastructure, (b) poor equipment, (c) little specialist training, and (d) economic constraints (endemic poverty of rural population). Alternatively, the remote population can use the rapid diagnostic test (RDT) provided by drug suppliers (step 7 in Fig. 5). RDTs, however, have several limitations (e.g. less accurate diagnosis). Data collected at the healthcare facility is analysed by spatial and decision support systems (SDSS) (step 2 in Fig. 5), computerized management systems aimed to smoothen complicated geographic issues [31]. These systems are usually based on a GIS platform and aim to back a better-informed decision of public health authorities (such as Ministry of Health) on malaria elimination (step 3 in Fig. 5).

An example of SDSS applied to malaria is represented by the system that has been developed in the South West Pacific archipelago to automatically locate and map the distribution of confirmed malaria cases, rapidly classify significant transmission centres, and guide targeted responses [32, 33]. As the SDSS relies on effective case detection, the performance of this system is dependent upon the quality of case reporting. However, healthcare facilities collect data not in real-time and with poor spatial accuracy [34]. Indeed, official records are likely to be linked to a health unit, a district, a municipality, or another level of spatial aggregation. Although data can be stored at the individual patient level, the spatial dimension is restricted to an aggregation that can hide crucial local diversity and thus hinder control efforts. Consequently, public health authorities cannot design efficient and flexible responses to malaria disease and cannot adequately direct the healthcare personnel (step 4 in Fig. 5). Moreover, doctors in healthcare facilities do not have a clear picture of transmission in the rural area because they are not onsite, and malaria testing offsite could be unreliable due to errors and inaccuracy. Therefore, they give directives to drug suppliers (step 5 in Fig. 5) to provide medicine to the population in an approximate way, resulting in inappropriate drugs delivery to the rural population and a waste of medical resources.

**Fig. 6:**
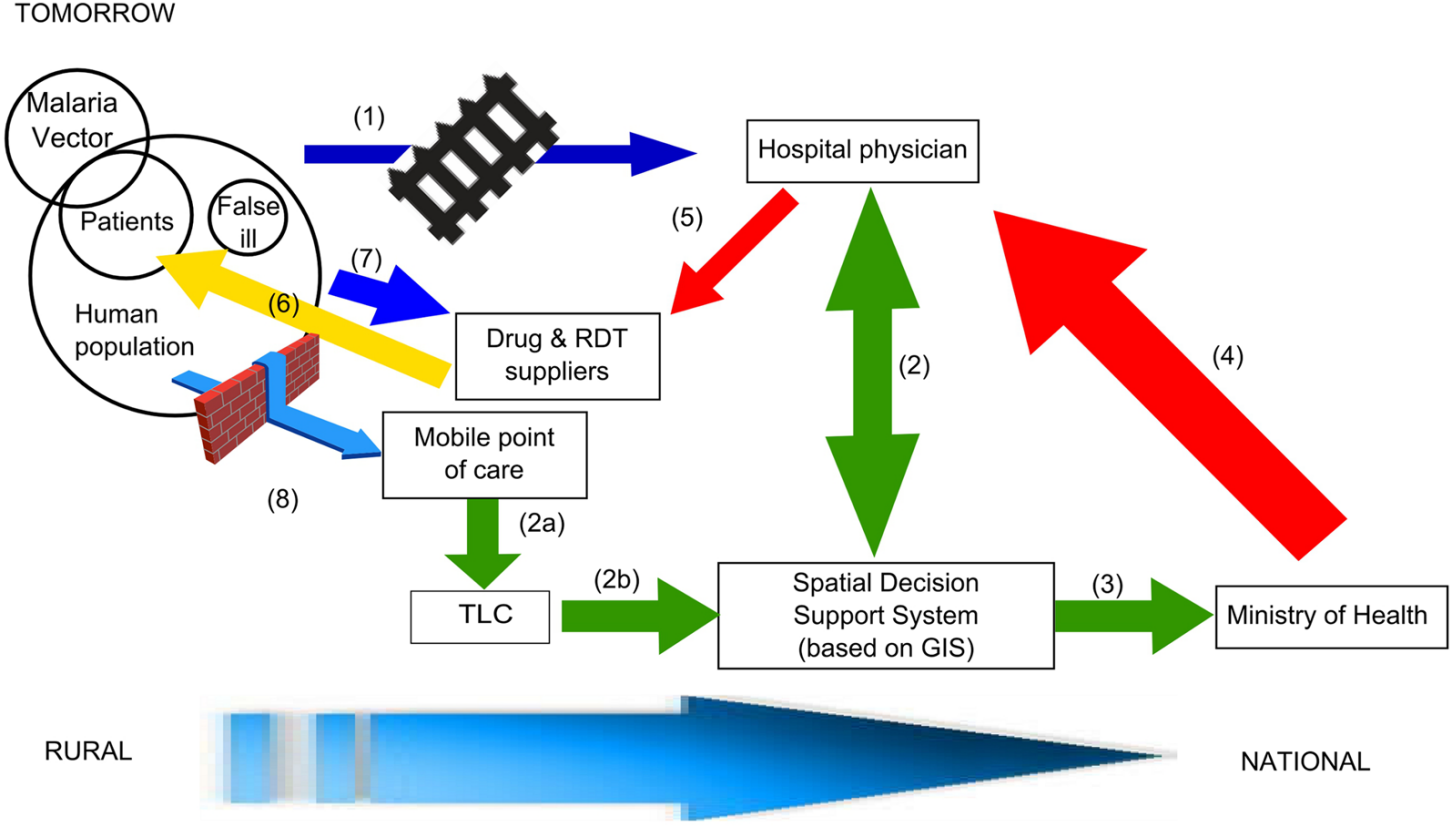
An hypothesis for a future malaria management process after the implementation of m-Health approach. Potential benefit of the introduction of m-Health approach and GIS to manage malaria in Uganda. *Blue arrows* represent physical movement; *red arrows* decision fluxes, *green arrows* information and data flow, and *yellow arrow* drugs supply. Size of the *arrows* gives a semi-quantitative measure of the increase of flow of people, material, data, information and decision. Each step (numbers in *brackets*) is described in the text

Figure 6 shows the same process with the innovative introduction of m-Health technologies integrated with GIS. The main innovation is the establishment of mobile points of care equipped with m-Health technologies (i.e. mobile microscopy). They help overcome some of the barriers obstructing the access of rural populations to proper malaria diagnosis and treatment (step 8 in Fig. 6). As a matter of fact, remote diagnosis helps to decongest health facilities where sick patients fuel malaria diffusion, and it significantly reduces costs and the challenges of timely transportation. In particular, in Uganda was proven that introduction of community healthcare services can reduce significantly the number of patient visits presenting as malaria and change the profile of cases seen at health facilities [35].

Moreover, the performance of any SDSS depends on a correct approach to key health system components, specifically at healthcare facilities and community levels [36]. The introduction of m-Health technology will automatically link case recording with the geographic position of the detection, almost in real time, limiting the discretion of the operators that record cases. This will help to overcome conventional SDSS challenges.

First, the m-Health/GIS integration will strengthen community engagement and malaria monitoring at local level encouraging initial treatment. As other scholars suggest vigilance at the community level is a best practice in the anti-malaria strategy in remote areas [37, 38].

Second, the diagnosis and case reporting will be more accurate, timely, and useful along the chain from local communities to healthcare structures, district authorities, and the Ministry of Health. To this extent, the preservation and safeguarding of the information flow will require the introduction of TLC stakeholders in the malaria management scheme (step 2a in Fig. 6).

Third, timely and accurate reporting (including geographical information) granted by m-Health technologies is a key factor for the effectiveness of any surveillance-response system. Indeed, the ability of health system components to geo-reference data and properly define cases influences the capability of the SDSS to routinely map the allocation of cases and precisely categorize malaria transmission hotspots.

The combination between m-Health technologies and GIS in an integrated SDSS could enable time and space shortcuts. The intensity of edge-connecting nodes (e.g. ill patients to diagnostic centres) depends on TLC signal quality (3G versus 2G standards). The integrated SDSS provides an efficient protocol to visualize and communicate the distribution pattern of malaria transmission a high degree of accuracy. Moreover, integrated GIS queries could enable malaria programme managers to place specific positive cases at a detailed (i.e. household) level; recognize, pick and map areas of main concern (e.g. malaria hotspot) prioritising the intervention; steer the choice of suitably focused, exclusive reaction; and dig out comprehensive additional statistics and facts.

The necessity of gathering more high quality data over a broad geographical area has already been indicated by malaria specialists in Uganda [25]. A network of sentinel sites (UMSN—Uganda malaria surveillance network) has been set up to collect data, ranging from malaria morbidity to mortality of carriers after exposure to insecticides. However, the UMSN is too small to allow a meaningful comparative analysis. The new technology proposed in this paper would allow creating a rich database from which patterns and trends could be identified through Big Data analysis. In order to gain a deeper understanding of the link between malaria and environmental, social and economical conditions it is, therefore, advisable to have a multidisciplinary approach to the subject, gathering data from fields such as climatology, entomology, education, genetics, geography, economics, and more. Big Data and GIS would help discover and visualize the emerging patterns and suggest a preferred course of action.

Assembling this comprehensive dataset facilitates rapid and efficient decision-making by public health authorities in marginal and remote areas and permits rapid and well-organized budget determination, resource allotment and workforce recruitment to aid the execution of actions within acknowledged disease hotspot (step 4 in Fig. 6). Initially, an RDT approach will be integrated into the new system (step 7 in Fig. 6) with the target to be substituted by mobile microscopy. Finally, the distribution of drugs will be enhanced and will be delivered to the appropriate patients avoiding the waste of resources on persons who are not sick (step 6 in Fig. 6).

Upgrading to 3G or 4G standards can substantially improve the efficacy of m-Health technologies. Network optimization exemplified in Fig. 6, softens location problems. Physical barriers will be partly overcome by technology, and organization will change sensibly. As a consequence:

- New stakeholders will emerge (TLC, software developers, GIS specialists, IT engineers);
- New equipment will be adopted;
- Information flows will be faster and more accurate across a wider network;
- Policy makers will have different data set on which to work (in real-time and more precisely geo-localized);
- Patients will have easier access to health services through mobile point-of-care;
- Drugs will be supplied, stored and utilized more efficiently;
- Prevention campaigns will be more targeted; legal experts will be involved in managing privacy issues arising from data transmission and cloud storage and computing.

### Technological and socio-economic aspects to be considered for implementing an integrated m-Health/GIS healthcare system

Considering the current trend to develop open source software for spatial analysis and geographic visualization, implementing such a system with those capabilities does not require significant financial investment on hardware or software, but only the human-resources investment to set up and maintain the system. A strategy of developing self-paced training packages that can be used to training individuals could possibly be used to customize some GIS software to minimize end-user skill requirements for its use [37].

The present study highlights also that the current 3G network is not sufficiently extended to support a mass application of m-Health technologies. Therefore, in remote areas where only the 2G mobile network is available, mobile microscopy could be conveniently used in offline mode if the Smartphone is equipped with automated cell-count software, which would enable field operators to diagnose malaria without a remote consultation through m-Health. In this case, the diagnosis would be instantly made on site at the remote point-of-care while the patient’s record would be transmitted to a larger health centre when faster (3G) connectivity is restored.

Future implementation of m-Health in the diagnosis and treatment of malaria should address some technological challenges:

- The majority of mobile phones in rural areas are “feature phones” (i.e. low-end phones with limited capabilities, often only 2G, with low computing capacity and limited memory). However, the cheap smart phones and apps will be deployed only for field operatives, with little investment. Should mobile microscopy be used by a larger number of people (for example enabling VHTs at village level to carry out tests—but need to consider training issues), then it would be necessary to upgrade from feature phone to Smartphone;
- Data transmission (e.g. sending a mobile microscope image from a village to a major health centre or hospital) can be difficult and slow without a 3G connection. Data compression is required, but this means a loss of data quality. Therefore, the development and testing of a suitable compression method represents a technological target;
- Data should be transferred in a readable format that could be integrated into the most widely used electronic health records to create, or integrate, a digital patient’s record.

An important concern is the quality of the data that governments have to manage and input in any disease control and surveillance system. Data transmission and management should be reliable, accurate, and safe, and at the same time the financial costs of m-Health/GIS system implementation should have the minimum impact on the poorest part of the population. If not properly addressed these issues will have three results:

1. Very remote locations lacking healthcare and ITC infrastructure will be more marginalized and underreported than now, cumulating the infrastructural (i.e. roads) and the digital divides;
2. If poor quality services are provided, people are likely to rely on local healers or ‘‘street doctors’’. Therefore, it is very important to take into consideration the social perception that population has of the m-Health technologies; and
3. The poorest may not have financial resources to pay for m-Health services and thus only seek care when severely ill [38].

Therefore, future research should focus not only technological aspects (mobile microscopy and GIS) of the innovation process but also on the social and economic impact of new technology on the stakeholders described in Fig. 6 (e.g. network analysis of stakeholders’ relationship).

Further refinement and validation of integration between SDSS and m-Health technologies should include a cost/benefit assessment of the surveillance systems. While the application of m-Health strategies involves initial costs, subsequent savings should rapidly bring to break-even and then to a positive payback.

Further studies to improve the effectiveness of the m-Health feasibility model should address healthcare facilities location—a central node for both the remote diagnosis and treatment. Identification of rural communities that have complicated access to the healthcare system is also crucial.

This study is limited to malaria in Uganda. However, exploring additional applications for various other vector-borne and infectious diseases such as dengue, filariasis, chikungunya, kala-azar, and Japanese encephalitis are very promising [39].

## Conclusions

This paper demonstrates the feasibility of an m-Health approach for malaria detection in a developing country (i.e. Uganda). The m-Health approach could have the largest impact, in terms of the remote population potentially involved in the system, in the west and the central-southeast regions of Uganda where m-Health could outreach the vastest portion of the remote population. In particular, six districts (Arua, Apac, Lira, Kamuli, Iganga, and Mubende) account for approximately 28% of the remote population affected by falciparum malaria with access to the 2G mobile network. This technology can improve access to low-cost, valuable and safe diagnostic protocol, making healthcare affordable even in forsaken environments. Further, malaria diagnosis in remote areas could help to decongest health facilities, reducing costs and contagion. Moreover, the combination of m-Health and GIS could provide real-time and geo-localized data transmission, enhancing anti-malaria strategies.

While empirical evidence of this study is limited to falciparum malaria in Uganda, wider applications, regarding other countries and pathologies, seem possible. Scalability can so be vertical and horizontal, geographically in other countries and concerning other diseases, such as TB or parasite-borne illnesses, separately or jointly with malaria.

Further research, to be conducted with an interdisciplinary approach, may comprehensively analyse in an innovative way classical topics such as:

- Integrated (and customized) medical history, through ICT recording of diagnosis and treatment;
- Digitalization of key information (malaria morbidity, healthcare/ICT spots, etc.) for big data processing;
- Just in time logistics, remembering that flexible m-Health networks may show much-wanted resilience when illnesses become pandemic;
- Healthcare facilities location that represents a central node for both the remote diagnosis and treatment, to improve the effectiveness of the m-Health feasibility model.
